# The cost-effectiveness of early noninvasive ventilation for ALS patients

**DOI:** 10.1186/1472-6963-5-58

**Published:** 2005-08-30

**Authors:** Kirsten L Gruis, Michael E Chernew, Devin L Brown

**Affiliations:** 1 Department of Neurology, University of Michigan Health System, Ann Arbor, Michigan, USA; 2 Department of Health Management and Policy, University of Michigan School of Public Health, Ann Arbor, Michigan, USA

## Abstract

**Background:**

Optimal timing of noninvasive positive pressure ventilation (NIPPV) initiation in patients with amyotrophic lateral sclerosis (ALS) is unknown, but NIPPV appears to benefit ALS patients who are symptomatic from pulmonary insufficiency. This has prompted research proposals of earlier NIPPV initiation in the ALS disease course in an attempt to further improve ALS patient quality of life and perhaps survival. We therefore used a cost-utility analysis to determine a priori what magnitude of health-related quality of life (HRQL) improvement early NIPPV initiation would need to achieve to be cost-effective in a future clinical trial.

**Methods:**

Using a Markov decision analytic model we calculated the benefit in health-state utility that NIPPV initiated at ALS diagnosis must achieve to be cost-effective. The primary outcome was the percent utility gained through NIPPV in relation to two common willingness-to-pay thresholds: $50,000 and $100,000 per quality-adjusted life year (QALY).

**Results:**

Our results indicate that if NIPPV begun at the time of diagnosis improves ALS patient HRQL as little as 13.5%, it would be a cost-effective treatment. Tolerance of NIPPV (assuming a 20% improvement in HRQL) would only need to exceed 18% in our model for treatment to remain cost-effective using a conservative willingness-to-pay threshold of $50,000 per QALY.

**Conclusion:**

If early use of NIPPV in ALS patients is shown to improve HRQL in future studies, it is likely to be a cost-effective treatment. Clinical trials of NIPPV begun at the time of ALS diagnosis are therefore warranted from a cost-effectiveness standpoint.

## Background

Respiratory failure is the most common cause of ALS patient death[1]. Prior to respiratory failure, respiratory muscle weakness can be measured by standard pulmonary function tests including forced vital capacity (FVC)[2]. Treatment of ALS patients with noninvasive positive pressure ventilation (NIPPV) when FVC is less than 50% appears to improve ALS patient survival[3,4] and quality of life [5-7]. Improved survival of ALS patients with NIPPV may be explained by a slower rate of pulmonary function decline[4,5].

NIPPV initiated early in the ALS disease course may off-load respiratory muscle work and thereby attenuate the progressive decrease in pulmonary compliance seen in ALS[8]. This treatment may also improve quality of life as ALS patients early in their course may experience non-specific symptoms of fatigue and lethargy, related to subtle respiratory muscle weakness, which goes unrecognized or is attributed to impaired mobility[2]. Whether initiation of NIPPV at diagnosis, when FVC is typically reduced but greater than 50%, slows the rate of pulmonary function decline and improves quality of life and survival remains to be studied in a clinical trial.

Paralleling proposed studies of feasibility and effectiveness of early NIPPV[9] is a need to determine what magnitude of health-related quality of life (HRQL) improvement this proposed treatment needs to achieve to be cost-effective. Quality of life improvement is an essential aspect of ALS treatment as curative treatments are not available[10]. The possible improvement in HRQL from early NIPPV treatment in ALS patients can be analyzed in conjunction with the costs of early NIPPV with a cost-utility analysis by using quality-adjusted life years (QALY) as a measure of effectiveness. Cost-utility analyses traditionally are used to determine which proven therapies are cost-effective by determining a treatment's incremental cost-effectiveness, that is, the cost per QALY gained relative to alternative treatments. In the "traditional" cost-utility analysis, effectiveness has been proven, and an estimate of benefit in health-state utility is already known. As society's willingness-to-pay costs per QALY have been reported,[11] the incremental cost-effectiveness can be compared to this standard to determine whether a newly proposed treatment is cost-effective. We applied this same process to determine a priori, how much benefit early NIPPV treatment in ALS patients would need to provide for this treatment to be cost-effective. We reasoned that should the degree of improvement determined in this analysis seem plausible, future clinical trials testing early NIPPV would be warranted from an economic perspective. If, on the other hand, the analysis showed that an impractical degree of improvement would be necessary for the treatment to be cost-effective, future clinical trials of early NIPPV for ALS would be less worthwhile.

## Methods

We calculated the benefit in health-state utility that early NIPPV treatment of ALS patients must achieve to be cost-effective. The primary outcome was the percent utility gained through NIPPV in relation to two common willingness-to-pay thresholds: $50,000 and $100,000 per QALY[11].

### Model

A decision tree modeled two alternative strategies: NIPPV starting at the time of diagnosis versus no NIPPV at the time of diagnosis, for a hypothetical cohort of patients with a recent diagnosis of ALS. Eighty percent of ALS patients have some evidence of respiratory muscle weakness at the time of initial diagnosis[12]; while half of patients demonstrate a reduction in FVC to less than 80% (approximately two standard deviations below the normal range) at initial presentation[13]. It was assumed that if early NIPPV is effective in preventing respiratory insufficiency, it should therefore be started at the time of diagnosis.

Patients were allowed to shift through disease states (mild, moderate, severe, terminal, or death) through Markov processes. The probabilities of patients progressing through these disease states over time were obtained from the literature[14]. All patients were modeled to begin in the mild stage, given their recent diagnosis. The Markov models used the average amount of time patients spend in each disease state, the probability of transitioning into a more severe stage of ALS, along with the utility associated with the time spent in each health state, to estimate the clinical and economic disease events over time. Per practice guidelines recommending the initiation of NIPPV based on an FVC < 50%, it was assumed that both groups would be treated with NIPPV when these criteria were met, and thus the analysis modeled only until this point. The time horizon used was 1 year as this is the average time period between diagnosis and meeting the NIPPV treatment criteria[3]. The reference case used a benefit in health-state utility of 20% in the NIPPV group compared with the non-NIPPV group. This is similar to the improvement in patient QOL demonstrated for NIPPV treatment in those who had respiratory muscle weakness, hypoventilation, or sleep-disordered breathing[7]. The improvement in health-state utility associated with NIPPV use was allowed to vary in sensitivity analysis, where one variable is allowed to vary over a plausible range. One-way sensitivity analyses were conducted for each variable across the ranges of values found in Table 1. To account for patients entering the model at varying rates of disease progression, the time horizon was adjusted. The time horizon was varied between 6 months and 2 years, in a one-way sensitivity analysis. As variations in FVC at entry may relate to ALS disease stage at entry, we also conducted a one-way sensitivity analysis on the probability of entering the model in the mild stage, as opposed to the moderate stage. Given that all patients are assumed to have recently been diagnosed with ALS and have an FVC > 50%, it was assumed that no one would enter the model in a severe or terminal state. The decision tree was analyzed by Data 4.0 (TreeAge Inc, Williamstown, MA).

**Table 1:** Probabilities, utilities, and costs for base case and sensitivity analysis

Variables	Reference case	Lower value tested	Upper value tested
Probabilities:			
NIPPV tolerance	0.49[3]	0.40	0.80
Staying mild	0.66[14]	0.60	0.70
Staying moderate	0.77[14]	0.70	0.80
Staying severe	0.76[14]	0.70	0.80
Staying terminal	0.78[14]	0.70	0.80
Utilities:			
Mild	0.8[15]	0.7	0.9
Moderate	0.6[15]	0.5	0.7
Severe	0.5[15]	0.4	0.6
Terminal	0.4[15]	0.3	0.5
Costs:			
NIPPV and accessories for 1 year	$3,132[17]	$2,810	$3,306
A trial of NIPPV in those who prove intolerant	$467[17]	$411	$483

### Utilities

Assessing health-state utilities in a patient population allows assignment of a numerical value to patient reports of HRQL or health state "utility" for different stages of disease. Health utilities were determined by assessment of patient's health state at each level of disease by a preference-based method and have been reported previously in control arms of large clinical trials[15]. These measurements were aggregated across individuals to determine utility scores for each health state, ranging from death (0), to perfect health (1). Utilities for each ALS stage, measured by the EuroQol EQ-5D visual analogue scale, were obtained from the literature[15].

### Costs

Costs were estimated from the Medicare fee schedule for 2004 (in US dollars) for NIPPV and NIPPV accessories. Medicare reimbursement was selected given the societal perspective[16]. Costs of one month of NIPPV rental and accessory costs were included for those intolerant to NIPPV. No discounting of costs or utilities was needed given the time horizon. Other costs related to ALS patient care were considered equal in both treatment groups given the identical probabilities of transitioning through health states and were therefore not entered into the model.

## Results

The average patient receiving NIPPV experienced 0.59 QALYs at a cost of $1,773; a patient not receiving NIPPV experienced 0.54 QALYs at a cost of $0, resulting in an incremental cost-effectiveness ratio of $33,801. Sensitivity analysis performed on the utilities of ALS states demonstrated NIPPV has an incremental cost-effectiveness ratio lower than $50,000 as long as the utility for ALS patients receiving NIPPV is at least 13.5% higher at each stage than those without NIPPV, meaning that early NIPPV is cost-effective as long as the treatment of ALS patients with NIPPV beginning at the time of diagnosis improves HRQL by at least 13.5%. For a willingness-to-pay threshold of $100,000 per QALY, the increase in HRQL with NIPPV would only need to be 6.8% or greater to be cost-effective.

The cost-effectiveness did not exceed the $50,000 willingness-to-pay threshold in any of the cost, transition probability, tolerance, or utility sensitivity analyses, meaning that alterations of each variable across a plausible range (Table 1) did not cause the incremental cost-effectiveness of NIPPV to exceed $50,000 per QALY. Altering the tolerance of NIPPV below 18% would however cause the cost-effectiveness to exceed $50,000 per QALY. No alteration of the probability of entering the model in the mild disease stage caused the incremental cost-effectiveness of NIPPV to exceed the $50,000 per QALY threshold. Shorter time horizons were associated with a lower cost-effectiveness ratio. A time horizon of 6 months was associated with an incremental cost-effectiveness of $76,909, while an 8 month time horizon was associated with incremental cost-effectiveness of $53,001. Time horizons of 10 months or above were associated with an incremental cost-effectiveness less than $50,000.

## Discussion

The benefit of early NIPPV use in ALS patients has not yet been studied. However, our cost-effectiveness model suggests that NIPPV begun at the time of diagnosis would be cost-effective if NIPPV were shown to improve HRQL by just 7–14%.

The 7–14% range of HRQL improvement that would be necessary in our model for early NIPPV to be cost-effective may be an overestimate. The $50,000 per QALY threshold for assessing cost-effectiveness is quite conservative. Given more recent estimates of the appropriate cost-effectiveness threshold[11], the improvement that would be necessary for NIPPV to be cost-effective is likely less than 7%. The possibility that NIPPV could slow the transition from less severe to more severe disease states was not taken into account in the model. Should early NIPPV be demonstrated to slow the progression of ALS[9], it would be even more cost-effective than this model suggests.

Tolerability of NIPPV by ALS patients with early disease is unknown. Tolerance of NIPPV (assuming a 20% improvement in HRQL) would only need to exceed 18% in our model for treatment to remain cost-effective using a conservative willingness-to-pay threshold of $50,000 per QALY. This estimate of NIPPV compliance is well lower than that seen in other studies[3]. The base case used a much more conservative estimate of 49% tolerance[3].

The current analysis was limited by the validity of the estimates used in the model. Tolerance of NIPPV administered early in the course of ALS is unknown, but this value was allowed to vary in sensitivity analysis. Utility values were ascertained from estimates in the literature, but previous studies on this topic are limited. The utility values were also allowed to vary in sensitivity analysis. In our model, early NIPPV remained cost-effective in all of the sensitivity analyses, supporting the robustness of the model.

## Conclusion

If early use of NIPPV in ALS patients is shown to improve HRQL in future studies, it is likely to be a cost-effective treatment. Further trials of early NIPPV initiation in ALS patients are warranted, and supported from a cost-effectiveness perspective.

## Competing interests

The authors declare(s) that they have no competing interests.

## Authors' contributions

KLG conceived the study, participated in its design and coordination and drafted the manuscript. MEC participated in the design of the study and helped with the statistical analysis and interpretation. DLB performed the statistical analysis, participated in the design of the study, and helped draft the manuscript. All authors read and approved the final manuscript.

## Pre-publication history

The pre-publication history for this paper can be accessed here:


